# A Rare Case of Myeloid Sarcoma Presenting as an Anorectal Ulcer

**DOI:** 10.1155/2012/537278

**Published:** 2012-05-17

**Authors:** Laxmi Parsa, Priti Bijpuria, Daniel Ringold, David Stein

**Affiliations:** Drexel University College of Medicine, Philadelphia, PA 19102, USA

## Abstract

Myeloid Sarcoma is a rare tumor composed of myeloblasts occurring at an extramedullary site like bones, or various soft tissues. Myeloid sarcoma may involve the gastrointestinal tract very rarely either solitarily, or occurring simultaneously with acute myeloid leukemia. Its diagnosis is challenging and needs biopsy and immunohistochemical staining. We are describing a case of myeloid sarcoma which presented as a painful anal ulcer mimicking an atypical fissure. Its appearance resembled crohn's disease on sigmoidoscopy. A biopsy of the ulcer along with histochemical staining led to the diagnosis of myeloid sarcoma. Our case demonstrates the need for aggressive evaluation of any common gastrointestinal complaint with an atypical presentation.

## 1. Background

Myeloid Sarcoma, also know as, “Chloroma,” “Granulocytic Sarcoma,” “Myeloblastoma,” or “extramedullary myeloid tumor,” is a tumor formed by immature myeloid cells or myeloblasts at an extramedullary site. Myeloid Sarcoma can precede the onset of acute myeloid leukemia and can also occur during active phase of leukemia. It may indicate the relapse of leukemia in a previously treated patient, or the blastic transformation of a chronic myeloid leukemia. Head and neck structures, bones, oral cavity, soft tissues, lymph nodes, skin, mediastinum, and reproductive organs are frequent sites of involvement of myeloid sarcoma. The involvement of the gastrointestinal tract is very rare. We report a unique case of myeloid sarcoma presenting as a large painful anorectal ulcer mimicking an anal fissure and Crohn's disease in a young pregnant woman.

## 2. Case Report

 A 30-year-old pregnant Caucasian female complaining of severe anal pain and an anal bump was referred to the Colorectal Surgery Office by her primary care physician. She was 8-week pregnant. Her pain started a few days earlier and she noticed a small amount of bright red blood on the toilet paper and coating the stools. She had been constipated. She stated the pain was worse during and after defecation. Her medical history was significant for irritable bowel syndrome, recurrent oral ulcers, and anal pruritis. She denied abdominal pain, nausea, or vomiting. Her family history was significant for inflammatory bowel disease in her maternal relatives. She denied any history of anal intercourse or instrumentation.

 Upon examination there were no significant physical findings with the exception of what appeared to be an atypical fissure with heaped up skin edges and ulceration in the center in the left posterior position. A local block was placed and a steroid injection was performed. Topical lidocaine mixed with diltiazem and high fiber supplementation were prescribed.

Her pain transiently improved but upon repeat examination two weeks later she was having recurrent pain and there was no change in the appearance of the fissure. She was admitted for pain control, an examination under anesthesia, and a flexible sigmoidoscopy. The differential diagnosis upon admission was new onset Crohn's Disease or a severe anal fissure. Upon admission, her vitals were normal and anal inspection revealed two large skin tags. A digital rectal exam could not be done secondary to severe tenderness. Labs showed normal serum electrolytes, WBC 3900, platelets 133000, Hb 11 g/dL, Hct 31.5, and MCV 91.

**Figure 1:**
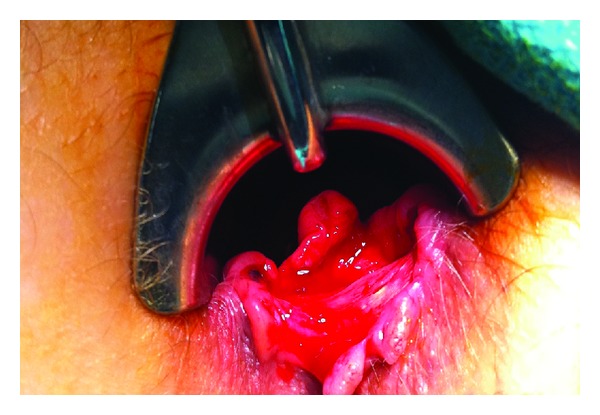
Large ano-rectal ulcer on exam under anesthesia.

She was taken to the operating room and examination demonstrated a large deep anal canal ulcer in the left posterior-lateral position (Figure 1). No obvious fistula or abscess was identified. A sigmoidoscopy was performed and revealed numerous aphthous erosions involving the mucosa of the rectum and sigmoid colon, which appeared consistent with inflammatory bowel disease. The proximal colon was not evaluated endoscopically. Biopsies were taken from the anal ulcer and rectosigmoid mucosa (Figure 3). An additional steroid injection was given into the ulcer bed. She was discharged home that day and was to follow up the following week. 

**Figure 2:**
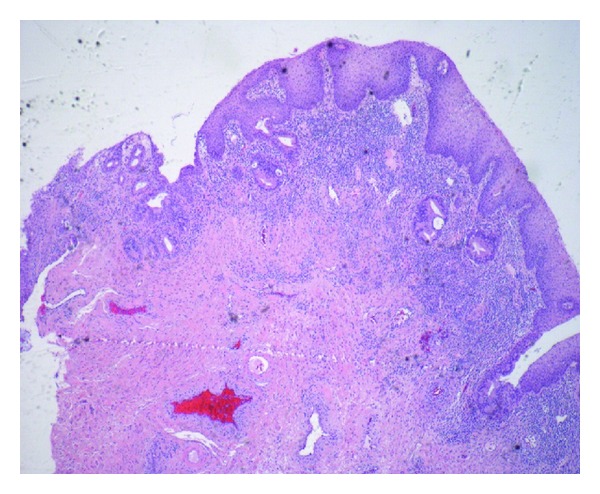
Rectal biopsy showing mucosal and submucosal atypical hematopoietic cell infiltration.

**Figure 3:**
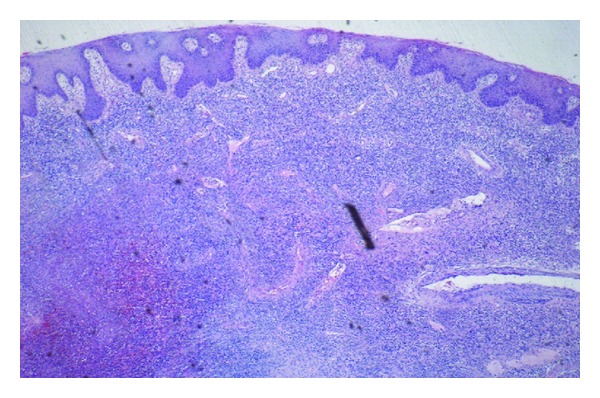
Anal ulcer biopsy showing atypical mucosal and submucosal hematopoietic cell infiltration.

**Figure 4:**
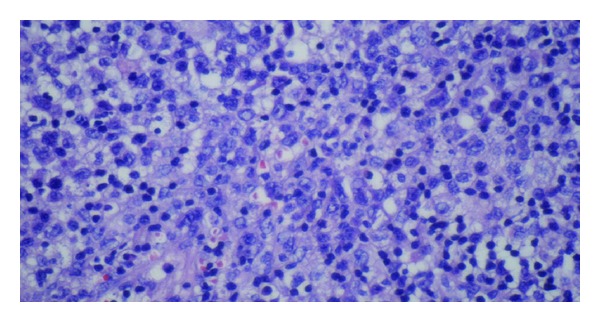
Rectal ulcer biopsy under high magnification shows atypical large and medium hematopoietic cells with large irregular nuclei and pale cytoplasm. Scattered small lymphocytes and plasma cells also seen.

**Figure 5:**
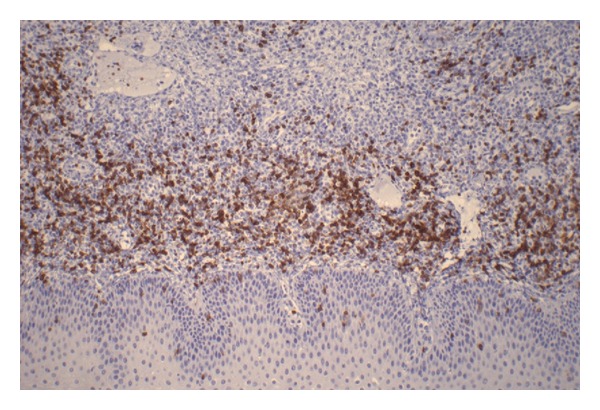
Immunohistochemical marker staining.

**Figure 6:**
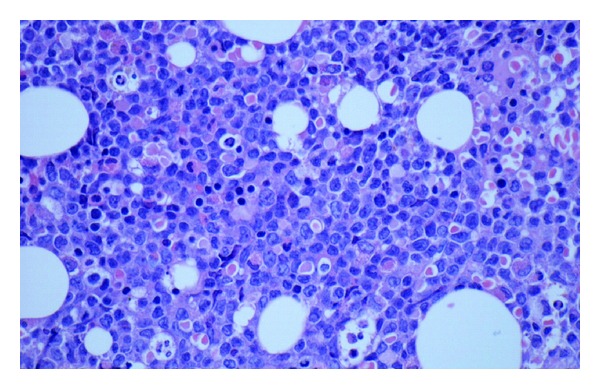
Bone marrow biopsy showing hypercellularity and atypical cells.

 In the following few days, the results of the peripheral blood smear and pathology returned. The smear contained 5% blasts. The biopsy from the anal ulcer depicted squamous mucosa with ulceration and submucosal atypical hematopoietic cell infiltrates suggestive of acute leukemia. Similar findings of discrete mucosal and submucosal aggregates of atypical large and medium-sized hematopoietic cells were noted on rectal biopsies. She was immediately readmitted and a bone biopsy was performed. Her results with flow cytometry showed hypercellularity with 53% blast cells. The abnormal cells were positive for CD13 (58%), CD33 (73%), CD15 (59%), CD45, CD34, CD64, CD4, HLA DR, and MPO markers consistent with acute monocytic leukemia (FAB M5b). Refer to Figures 2, 4, 5 and 6. Subsequent cerebrospinal fluid analysis also showed 30% blasts on flow cytometry. Her cytogenetic evaluation revealed an abnormal female karyotype with Inv (16) (p13q22), and Trisomy 8 and 22.

She was diagnosed with acute myeloid leukemia and started on high-dose Idarubicin and cytarabine for induction chemotherapy. Intrathecal methotrexate was also given as there was evidence of central nervous system disease. Her pregnancy was terminated therapeutically before the chemotherapy was started. A repeat bone marrow biopsy nine months later after completing chemotherapy did not show any atypical cells. She clinically has no evidence of disease at this time.

## 3. Discussion

 Myeloid sarcoma is a tumor composed of immature myeloid cells or myeloblasts at an extramedullary site often in close proximity to bone. Myeloid sarcoma can occur as a solitary mass or multiple nodules in different tissues or organs such as skin, bone, head and neck structures, oral cavity, gingiva, salivary glands, lymph nodes, central nervous system, and genital tract, and rarely in the gastrointestinal tract [1, 2]. Myeloid sarcoma can occur concurrently with acute myeloblastic leukemia in advanced stages. Skin and mucosa are the most common sites of involvement. Sometimes, myeloid sarcoma can be the initial presentation of blastic transformation of a chronic myeloproliferative disorder or a myelodysplastic syndrome [1, 3]. It has also been described as a manifestation of relapse in patients with history of prior successful treatment of acute or chronic leukemias after bone marrow and stem cell transplantation [1, 4]. Isolated myeloid sarcoma is rarely diagnosed in the absence of acute myeloblastic leukemia. A diagnosis of acute myeloblastic leukemia will be typically made a few months to years after the diagnosis of myeloid sarcoma. The prognosis is poor [5, 6].

 The involvement of the gastrointestinal tract in different hematologic malignancies occurs at an advanced stage of the disease or as a complication of chemotherapy and radiotherapy. The most common findings on endoscopy includes esophagitis, colitis, gastritis, gastric erosions, duodenitis, proctitis, and polyps [7]. Myeloid sarcoma involving the gastrointestinal tract is very rare and can present with abdominal pain, bleeding, perforation, obstruction, intussusception, liver infarction, pancreatitis, appendicitis, bile duct obstruction, and portal hypertension [8–11]. CT imaging might show focal bowel wall thickening, a single mass, polyps, multiple masses obstructing the gut lumen, or exophytic lesions involving the peritoneum [12]. There have been cases of myeloid sarcoma localized to the oral cavity [13], esophagus, stomach, jejunum [14–16], gallbladder [17], bile ducts [18], pancreas [19, 20], colon [21], liver [21], or appendix [22] or may diffusely involve the GI tract [23–25] including the abdominal wall [26]. When small and large bowels are involved, it is more likely to spread to the mesentery and peritoneum. The frequent involvement of the ileum, appendix, and colon is likely secondary to the widespread distribution of lymphoid tissue [8]. Our patient has involvement of anus, rectum, and sigmoid colon.

 Myeloid sarcoma poses a significant diagnostic challenge. Biopsy and staining with immunohistochemical markers play a vital role. The immunophenotype is characteristic based on whether myeloid sarcoma is granulocytic (MPO+, Lysozymes+, CD34+/−), monoblastic (MPO−, CD68+, Lysozyme−, CD34+), myelomonoblastic (MPO+/−, cd68+, lysozyme+/−, CD34+/−), megakaryoblastic (Factor VIII+, CD31+), or erythroblastic variant (glycoprotein c+) [1, 27]. It is difficult to distinguish myeloid sarcoma from medium sized or large cell non-Hodgkin's lymphoma. In addition, small round cell tumors, undifferentiated carcinomas, and extramedullary localizations of chronic myeloproliferative disorders also present significant confusion [1, 27] during diagnosis. The most frequent chromosomal abnormality is *t* [8, 21] which our patient did not have. Patients with an acute myeloblastic leukemia associated with *t* [8, 21] and presenting with myeloid sarcoma have a low rate of complete remission, and overall survival is poor [1, 28, 29]. Myeloid sarcoma is treated with chemotherapy and local radiation therapy when appropriate even there is no evidence of AML on bone marrow evaluation.

 The patient in this report presented with anal pain which was initially presumed to be a fissure and treated accordingly. Her history of irritable bowel syndrome and her family history of inflammatory bowel disease gave the clinical impression of Crohn's Disease with an atypical anal ulcer. An aggressive workup leading to admission and an examination under anesthesia led to the “confirmatory” diagnosis of anorectal Crohn's Disease. The smear and histopathology with immunohistochemical markers diagnosed the myeloid sarcoma. A subsequent bone marrow evaluation, CSF analysis with flow cytometry, and cytogenetic studies confirmed the AML to allow initiation of treatment.

 We did not find any association of pregnancy with increased incidence of myeloid sarcoma or acute myeloid leukemia in our review. Though, myeloid sarcoma was reported to involve the female genital tract [30–32] and pregnancy is associated with rapid maternal hematopoiesis, the risk of acute myeloid leukemia in pregnancy is relatively very rare [33, 34]. We came across only one case report of myeloid sarcoma occurring in a pregnant woman [35] previously. The patient in above case discussion was diagnosed with myeloid sarcoma and AML in her first trimester and her pregnancy was terminated therapeutically.

It is unusual to find a diagnosis of myeloid sarcoma and AML with patients who present with common gastrointestinal problems. Our case illustrates the need for aggressive evaluation in any patient presenting with atypical gastrointestinal complaints.
